# Plasma NfL, ptau181 and GFAP as potential biomarkers for neuropsychiatric symptoms and related long‐term outcomes in a memory clinic cohort

**DOI:** 10.1002/alz.092191

**Published:** 2025-01-09

**Authors:** Miriam Rabl, Leonardo Zullo, Armin von Gunten, Julius Popp

**Affiliations:** ^1^ Psychiatric University Clinic Zurich and University of Zurich, Zurich Switzerland; ^2^ Lausanne University Hospital, Lausanne Switzerland; ^3^ University of Zurich, Zurich Switzerland

## Abstract

**Background:**

Neuropsychiatric symptoms (NPS) are common in older people, may occur early in developing dementia, and have been associated with worse long‐term outcomes. The objectives here were to investigate whether plasma neurofilament light chain (NfL), tau phosphorylated at threonine 181 (ptau181), and glial fibrillary acid protein (GFAP) are associated with a) current NPS, b) future NPS, c) NPS severity change and d) cognitive and functional decline over time. And whether the presence of NPS combined with plasma biomarkers are useful to predict these long‐term outcomes (b‐d).

**Method:**

151 participants with normal cognition (n=67) or cognitive impairment (mild cognitive impairment and mild dementia, n=84) were examined in a longitudinal memory clinic study. Plasma NfL, ptau181 and GFAP were measured at baseline. NPS were assessed through the Neuropsychiatric Inventory Questionnaire (NPI‐Q) at baseline and follow‐up, while cognitive and functional decline was evaluated trough the Clinical Dementia Rating – sum of boxes (CDR‐SB) score (mean: 22 months). Linear regression and ROC analysis were used to address the associations of interest.

**Result:**

Cross‐sectionally none of the three plasma biomarkers was associated with NPS after adjusting for age, sex and cognitive impairment. Higher NfL was related to NPS at follow‐up (β = 0.24, 95% CI 0.04‐0.45, p = .022). NfL (β = 0.22, 95% CI 0.00‐0.44, p = .047) and GFAP (β = 0.26, 95% CI 0.05‐0.48, p = .017) were associated with an increase in the NPI‐Q severity at follow‐up. Furthermore, both markers were associated with increase in the CDR‐SB score (NfL: β = 0.29, 95% CI 0.06‐0.52, p = .013; GFAP: β = 0.33, 95% CI 0.11‐0.56, p = .004). Adding NPS and all three plasma biomarkers to a reference model improved the prediction of future NPS (AUC 0.75 to 0.84, p = .015) and cognitive decline (AUC 0.87 to 0.95, p = .030) but not the change in the NPI‐Q severity (AUC 0.67 to 0.71, p = .264) over time.

**Conclusion:**

NfL and GFAP are associated with NPS and related long‐term outcomes. Considering the presence of NPS along with blood‐based biomarkers may improve prediction of clinical progression in memory clinic patients and improve clinical decision making.

